# Differential analysis of serum immunology and gut microbiota in patients with gastrointestinal diseases

**DOI:** 10.3389/fmicb.2024.1323842

**Published:** 2024-05-01

**Authors:** Huan Zhu Chen, Yu Yang Zeng, Guo Xiong Cai, Wei Dan Gu, YaLi Yang

**Affiliations:** ^1^Biochemistry Teaching and Research Office of the Basic Department of the Medical College of Jiaying University, Meizhou, China; ^2^Laboratory Department of the Affiliated Hospital of the Medical College of Jiaying University, Meizhou, China; ^3^Guangdong Provincial Key Laboratory of Conservation and Precision Utilization of Characteristic Agricultural Resources in Mountainous Areas, Meizhou, China

**Keywords:** gastrointestinal diseases, serum, immunology, gut, microbiota

## Abstract

**Objective:**

Gastric and intestinal diseases possess distinct characteristics although they are interconnected. The primary objective of this study was to investigate the pathogenesis of gastrointestinal diseases through different analyses of clinical characteristics, serum immunology, and gut microbiota in patients with gastrointestinal diseases.

**Methods:**

We collected serum samples from 89 patients with gastrointestinal diseases and 9 healthy controls for immunological assessment, stool samples for DNA extraction, library construction, sequencing, as well as clinical data for subsequent analysis.

**Results:**

Regarding clinical characteristics, there were significant differences between the disease group and the healthy control (HC) group, particularly in terms of age, cancer antigen 125 (CA125), cancer antigen 199 (CA199), alpha-fetoprotein (AFP), total bilirubin (TBIL) and indirect bilirubin (IBIL). The intestinal disease (ID) group exhibited the highest IL-6 level, which significantly differed from the stomach disease (SD) group (*p* < 0.05). In comparing the HC with the ID groups, significant differences in abundance were detected across 46 species. The HC group displayed a greater abundance of *Clostridiales, Clostridia, Firmicutes, Bifidobacterium, Bifidobacteriaceae, Bifidobacteriales, Actinobacteria, Veillonellaceae, Longum, Copri, Megamonas* and *Callidus* than other species. Similarly, when comparing the HC with the SD groups, significant differences in abundance were identified among 49 species, with only one species that the *Lachnospiraceae* in the HC group exhibited a higher abundance than others. Furthermore, certain clinical characteristics, such as CA125, CA199, glucose (Glu), creatine kinase-MB (CKMB) and interleukin-22 (IL-22), displayed positive correlations with enriched gut species in the ID and SD groups, while exhibiting a negative correlation with the HC group.

**Conclusion:**

The disturbance in human gut microbiota is intimately associated with the development and progression of gastrointestinal diseases. Moreover, the gut microbiota in the HC group was found more diverse than that in the ID and SD groups, and there were significant differences in microbial species among the three groups at different classification levels. Notably, a correlation was identified between specific clinical characteristics (e.g., CA125, CA199, Glu, CKMB and IL-22) and gut microbiota among patients with gastrointestinal diseases.

## 1 Introduction

The human gastrointestinal (GI) tract contains a rich microbial community, aggregating about 100 trillion microbes. In addition to bacteria and fungi, the human gut microbiota also includes viruses, bacteriophages and archaea. The gut microbiota is considered as one of the most important types of microorganisms for maintaining human health (Hou et al., 2022a), such as food fermentation, protecting the body from pathogenic harm, stimulating immune responses and producing vitamins (Heintz-Buschart and Wilmes, 2018; Adak and Khan, 2019; de Vos et al., 2022). Regarding the immune system, the human microbiota can not only protect the host from external pathogens by producing antibacterial substances, but also play a significant role in the development of intestinal mucosa and immune system (Sittipo et al., 2018). Thus, the maturation of the immune system requires the development of symbiotic microbes while the GI tract has numerous immune cells. For example, one mechanism by which gut microbiota affects the immune system is mediating neutrophil migration, thereby influencing T cell differentiation into diverse types, such as helper T cells (Th1, Th2, and Th17 cells) and regulatory T (Treg) cells (Wu et al., 2021; Shim et al., 2023). Interestingly, the composition and diversity of gut microbiota can predict responses to immunotherapy with immune checkpoint inhibitors (ICIs) (Morkunas et al., 2020). For instance, gut microbiota can affect adoptive T-cell transfer (ACT) immunotherapy strategies, CpG oligodeoxynucleotide (CpG ODN) immunotherapy strategies and cell-based immunotherapies by regulating innate immunity, adaptive immunity and tumor antigens to improve ICI response (Lu et al., 2022). Additionally, adaptive immune response is another important part of maintaining a healthy microbiota and immune balance, achieved through the differentiation and maturation of B and T cells, as well as the establishment of immune tolerance to the microbiota. According to the type of bacteria, CD4T cell responses vary greatly, leading to differentiation into different subgroups and subsequent release of pro-inflammatory cytokines, such as interferon-γ and interleukin-17A (IL-17A) (Mazmanian et al., 2005; Weaver et al., 2006; Alnahas et al., 2017).

To date, a growing body of evidence has confirmed that the microbiota is associated with the development of cardiovascular diseases (Lau et al., 2017; Sanchez-Rodriguez et al., 2020), cancer (Kostic et al., 2013; Jin et al., 2019; Sami et al., 2020; Vyhnalova et al., 2021), respiratory diseases (Segal et al., 2013; Bassis et al., 2015; Cait et al., 2018; Sokolowska et al., 2018), diabetes (Larsen et al., 2010; Vatanen et al., 2018; Hou et al., 2022b), inflammatory bowel disease (IBD) (Manichanh et al., 2012; Ahmed et al., 2016; Lucas Lopez et al., 2017), brain diseases (de Theije et al., 2014; Gilbert, 2015; Allen et al., 2016; Zhang et al., 2022), chronic kidney disease (Chung et al., 2019; Lun et al., 2019; Pluznick, 2020), liver disease (Mouzaki et al., 2013; Tripathi et al., 2018) and osteoporosis (Qin et al., 2021). Thus, the manipulation of human microbiota may be the key to disease treatment. It is noteworthy that the relative distribution of microbes within the gut is individual-specific and can even vary within the same individual (Hou et al., 2022a). Numerous factors have been identified as regulators of the host’s gut-brain axis, both internally and externally, encompassing genetics, socioeconomic status, dietary habits, medication use and environmental influences.

Regarding cancers, *Helicobacter pylori* is regarded as a pathogenic factor in gastric cancer (Hu et al., 2022), while *Fusobacterium nucleatum* is recognized as a contributing factor in colorectal cancer (CRC) (Bullman et al., 2017). *Escherichia coli* strains that possess Polyketide synthase (PKS) genotoxins have the potential to promote colorectal tumorigenesis. *Bacteroides fragilis*, on the other hand, can generate reactive oxygen species (ROS), which can lead to DNA damage. Although these bacteria have a direct impact on the occurrence of tumors, some microorganisms can promote inflammation or weaken immune surveillance, indirectly promoting the development of cancer. These microbial immune regulatory activities are known as the immune-tumor-microbe axis (Ting et al., 2022). Gut microbiome can induce epithelial barrier deterioration, triggering tumor-induced inflammation, as well as driving progression of CRC through influencing certain signaling pathways (e.g., E-cadherin/β-catenin, TLR4/MYD88 and SMO/RAS/p38MAPK) (Li et al., 2021). The elevated expression levels of circular RNAs, specifically circRNA ciRS-7, circNSUN2 and circ-ERBIN, have been identified to be contributed to the progression of CRC. Meanwhile, CircIL4R has been found to enhance the proliferation, migration and invasion of CRC cells by modulating the PI3K/AKT signaling pathway (Jiang et al., 2021). In addition to bacteria and fungi, viruses exhibit distinctive disease characteristics within CRC. The presence of viruses indirectly influences CRC by altering the associated bacterial ecosystem (Li et al., 2022).

Gut microbiota is highly associated with the development of IBD. Mechanistically, dysbiosis of the microbiota is correlated with IBD through influencing inflammation and intestinal barriers (Konig et al., 2016). As reported by Kleessen et al. (2002), a higher prevalence of mucosal bacterial infiltration was found in the IBD group compared to the control. Notably, in patients with Crohn’s disease (CD), there was a significant elevation in the presence of adherent-invasive *E. coli*, indicating that pathogens could influence intestinal permeability, the composition of the gut microbiota and ultimately contribute to intestinal inflammation (Ahmed et al., 2016). The pathophysiology of IBD is multifactorial, involving complex interactions among genetics (Marcuzzi et al., 2013), environment (Leone et al., 2013), microbiome (Manichanh et al., 2012; Wang et al., 2014), immunity (Kaistha and Levine, 2014) and potential other risk factors.

There are many existing analyses on the differences in gut microbiota among patients with gastrointestinal diseases, but there are few analyses on the differences in gut microbiota among patients with gastric diseases, and there is a lack of analysis on the differences in serum immunology and gut microbiota among patients with gastrointestinal diseases, which hinders further research on the role of microbiota. Although stomach and intestinal diseases share some common risk factors, they have different phenotypes and disease manifestations. This cross-sectional cohort study analyzed the clinical characteristics, serum immunology and differences in gut microbiota of patients with gastrointestinal diseases, aiming to explore the pathogenesis of gastrointestinal diseases, further prevent and reduce the risk of gastrointestinal diseases in China through customized intervention measures, and develop personalized multimodal treatment strategies.

## 2 Materials and methods

### 2.1 Collection of samples and clinical data

Serum and fecal samples: serum and fecal samples were collected from 89 patients with gastrointestinal diseases who were admitted to the Affiliated Hospital of Jiaying Medical College (Meizhou, China, which was established in November 2009 and is the first university affiliated hospital in Meizhou City. It is under the management of Jiaying College Medical College and is a public non-profit comprehensive hospital) from March 2021 to March 2022, and 9 healthy individuals were also involved as healthy controls. A total of 500 μL of serum samples were collected in 2 mL sterile centrifuge tubes, while 50 g of fecal samples were gathered in specialized fecal collection tubes. These samples were subsequently stored at −80°C for future utilization.
Inclusion criteria for patients with gastrointestinal diseases: patients diagnosed with stomach or/and intestinal diseases (such as inflammation, tumors, functional disorders, etc.). Exclude patients with other tumors, liver disease, kidney disease or other traumatic bleeding at the same time.
Inclusion criteria for the healthy control group: volunteers who have passed physical examinations and do not have any stomach or/and intestinal diseases, tumors, liver diseases, kidney disease or other traumatic bleeding are recognized as healthy.
Clinical data: patients’ and healthy controls’ data, including date of admission, age, gender, routine blood test results and biochemical indicators were recorded.

### 2.2 Immunological tests

The levels of relevant cytokines, including IL-6, IL-9 and IL-22 in the serum, were determined using human interleukin 6, 9 and 22 ELISA kits (Jiangsu Meimian, China). Additionally, the levels of cytokines, such as IL-8, IFN-γ and TNF-α were measured using human interleukin 8, IFN-γ and TNF-α ELISA kits (Guangzhou Daan, China). The specific procedures for these assessments were carried out in accordance with manufacturers’ instructions.

### 2.3 DNA extraction, DNA library construction and sequencing of fecal samples

Among 89 patients with gastrointestinal diseases, 16S rDNA amplifier sequencing was performed on 4 patients with gastric diseases, 17 patients with intestinal diseases and 9 individuals undergoing routine medical examinations. Universal primers designed for conserved regions were utilized for PCR amplification, followed by double-end (Paired-End) sequencing on the MISEQ/Hiseq platform, generating one or more high-variability 16S rDNA sequences. These sequences were then subjected to operational taxonomic unit (OTU) clustering, species annotations and abundance analysis, along with assessments of α- and β-diversity, as well as other analyses.

Total DNA extraction from the microbial community in stool samples was carried out using the E.Z.N.A^®^ Stool DNA kit (Omega Bio-tek, Norcross, GA, United States), and the DNA was quantified using a Thermo NanoDrop 2000 (Thermo Fisher Scientific, Waltham, MA, United States).
For PCR amplification, the target sequence was amplified with specific primers (341F: 5′-CCTACGGGNGGCWGCAG-3′, 805R: 5′-GACTACHVGGGTATCTAATCC-3′). The resulting PCR product was confirmed through electrophoresis on a 2% agarose gel. Subsequently, PCR products were purified using AMPure XT beads (Beckman Coulter Genomics, Danvers, MA, United States) and quantified with Qubit (Invitrogen, Carlsbad, CA, United States). The amplicon pool was utilized for sequencing. The size and quantity of the amplified library were assessed via the Agilent 2100 Bioanalyzer (Agilent Technologies Inc., Santa Clara, CA, United States) and the Illumina Library Quantitative Kit (Kapa Biosciences, Woburn, MA, United States). Sequencing of the library was conducted on the Illumina NovaSeq PE250 platform (Illumina Inc., San Diego, United States).

### 2.4 Statistical analysis

Statistical analysis was performed using SPSS 22.0.0.0.202 software (IBM, Armonk, NY, United States). Descriptive statistics for continuous variables following a normal distribution were expressed as mean ± standard deviation (SD). Multivariate analysis of variance (ANOVA) was utilized to compare multiple samples. In cases of abnormally distributed continuous variables, descriptive statistics were presented as median and interquartile range (IQR), and the Mann–Whitney *U* test was employed for making comparisons between two groups. Variations of species between groups were analyzed using *t*-tests, nonparametric rank sum tests (*U* tests), and linear discriminant analysis effect size (LEfSe). The Analysis of similarities (ANOSIM) (Chapman and Underwood, 1999) was applied to assess differences in community structure between groups, and Spearman’s rank correlation was utilized to evaluate associations between microbial diversity and biochemical indicators. The significance level was set at 5%.

## 3 Results

### 3.1 Comparison of clinical characteristics between the two groups

According to the specific type of disease, 89 patients were categorized into distinct groups, namely the stomach disease (SD) group consisting of 13 cases, the intestinal disease (ID) group encompassing 60 cases, the gastrointestinal disease (GD) group comprising 16 cases, and healthy control (HC) group involving 9 cases. In terms of age, the mean age of the HC group was the youngest at 33.11 years old, which was notably different from that in the SD and ID groups (*p* < 0.01). When comparing the results of routine blood tests and biochemical indicators, it was noted that the levels of cancer antigen 125 (CA125) and cancer antigen 199 (CA199) in the HC group were significantly different from those in the three disease groups (*p* < 0.01). The median levels of CA125 and CA199 were the lowest in the HC group (1.21, 3.28), with the highest median level of CA125 observed in the SD group (19.50) and the highest median level of CA199 found in the GD group (29.04). There was also a significant difference in CA125 level between the SD group and the ID & GD groups (*p* < 0.01). Alpha-fetoprotein (AFP) yielded its highest median level in the GD group (4.44), demonstrating a highly significant difference compared with the SD & HC groups (*p* < 0.01). Similarly, the highest mean neutrophil (N) count was identified in the GD group (7.27), which exhibited a significant difference compared with the SD group (*p* < 0.05). Total bilirubin (TBIL) and indirect bilirubin (IBIL) reached their highest mean levels in the SD group (15.64, 10.35), demonstrating significant differences compared with the HC group (*p* < 0.05). Regarding other characteristics, no significant differences were found among all the groups (Table 1).

**Table 1:** Main demographic and serological characteristics in disease groups and healthy control group.

	SD	ID	GD	HC
Cases	13	60	16	9
Gender (F/M)	6/7	27/33	7/9	4/5
Age	68.15 ± 12.36^#^	57.47 ± 17.44^■^	41.06 ± 20.60	33.11 ± 4.17
CA125 (U/mL)	19.50 (12.23, 35.24)^#▲^	12.87 (10.56, 14.55)^*■^	13.06 (9.51, 16.21)^△^	1.21 (1.00, 1.85)
CA199 (U/mL)	20.80 (15.33, 33.47)^#^	19.18 (9.43, 29/04)^■^	29.04 (9.90, 29.04)^△^	3.28 (1.34, 7.81)
AFP (ng/mL)	2.84 (2.08, 3.18)^▲^	3.86 (2.67, 4.65)	4.44 (3.94, 4.84)^△^	2.94 (2.78, 3.11)
CEA (ng/mL)	3.07 (2.10, 3.59)	2.61 (1.27, 4.20)	2.15 (1.30, 3.90)	3.54 (2.24, 5.23)
CK (U/L)	123.08 ± 33.58	132.72 ± 51.50	137.56 ± 50.85	121.89 ± 40.93
CK-MB (U/L)	26.15 ± 10.21	29.92 ± 19.00	33.44 ± 20.59	20.67 ± 8.22
LDH (U/L)	182.69 ± 27.91	185.30 ± 31.94	187.56 ± 24.82	195.89 ± 34.93
AST (U/L)	23.00 (11.50, 27.00)	23.35 (18.00, 26.00)	22.00 (17.75, 24.00)	20.00 (15.00, 24.50)
ALT (U/L)	14.00 (12.00, 24.50)	21.00 (16.00, 26.00)	17.00 (14.25, 22.00)	16.00 (13.00, 22.00)
GGT (U/L)	24.00 (14.50, 34.50)	25.00 (18.25, 30.83)	24.00 (15.00, 45.00)	36.00 (12.50, 37.00)
CHE (U/L)	6843.08 ± 1901.03	8044.77 ± 1277.10	8571.81 ± 1753.84	5918.78 ± 2640.70
TP (g/L)	71.00 (64.50, 76.50)	72.00 (71.00, 74.00)	72.00 (68.00, 72.00)	72.00 (72.00, 75.50)
ALB (g/L)	43.00 (41.50, 47.50)	45/00 (44.00, 47.00)	44.00 (42.00, 46.00)	43.00 (43.00, 47.50)
TBIL (μmol/L)	15.64 ± 11.63^#^	12.14 ± 3.67	12.90 ± 6.25	11.54 ± 2.05
DBIL (μmol/L)	5.68 ± 3.74	4.90 ± 1.72	4.90 ± 1.42	4.72 ± 0.98
IBIL (μmol/L)	10.35 ± 7.82^#^	7.41 ± 2.30	7.36 ± 4.40	7.32 ± 1.35
Glu (mmol/L)	7.63 ± 4.58	6.33 ± 1.10	6.42 ± 1.65	5.69 ± 0.85
WBC (×10^9^/L)	5.73 (4.78, 8.07)	6.90 (5.36, 9.27)	8.52 (6.40, 12.47)	6.56 (4.45, 8.00)
HB (g/L)	128.15 ± 32.36	128.44 ± 21.81	134.81 ± 22.47	139.56 ± 14.93
PLT (×10^9^/L)	220.77 ± 77.27	248.58 ± 76.51	242.88 ± 63.88	270.33 ± 43.96
N (×10^9^/L)	4.00 ± 1.87^▲^	5.20 ± 3.24	7.27 ± 3.82	5.07 ± 2.04
LY (×10^9^/L)	1.46 (1.30, 1.91)	1.71 (1.25, 2.12)	1.36 (0.93, 1.76)	1.69 (1.51, 2.05)
M (×10^9^/L)	0.45 (0.31, 0.58)	0.51 (0.41, 0.95)	0.51 (0.36, 0.75)	0.49 (0.43, 1.11)
E (×10^9^/L)	0.09 (0.05, 0.27)	0.10 (0.04, 0.17)	0.07 (0.02, 0.19)	0.11 (0.08, 0.12)
B (×10^9^/L)	0.02 (0.01, 0.04)	0.02 (0.01, 0.03)	0.02 (0.01, 0.03)	0.02 (0.02, 0.05)

Explanatory note: main demographic and serological characteristics in disease groups and healthy control group, there were significant differences in age, cancer antigen 125 (CA125), cancer antigen 199 (CA199), alpha fetoprotein (AFP), mean neutrophil count (N), total bilirubin (TBIL), and indirect bilirubin (IBIL) during comparison. F, female; M, male; CA125, cancer antigen 125; CA199, carbohydrate antigen 199; AFP, alpha-fetoprotein; CEA, carcinoembryonic antigen; CK, creatine kinase; CK-MB, creatine kinase isoenzyme MB; LDH, lactate dehydrogenase; AST, aspartate aminotransferase; ALT, alanine aminotransferase; GGT, gamma-glutamyl transferase; CHE, cholinesterase; TP, total protein; ALB, albumin; TBIL, total bilirubin; DBIL, direct bilirubin; IBIL, indirect bilirubin; Glu, glucose; WBC, white blood cells; Hb, hemoglobin; PLT, platelets; N, neutrophils; LY, lymphocytes; M, monocytes; E, eosinophils; B, basophils. ^*^p < 0.01 when SD is compared with ID; ^▲^*p* < 0.01 when SD is compared with GD; ^#^*p* < 0.01 when SD is compared with HC; ^■^*p* < 0.05 when ID is compared with HC; ^△^*p* < 0.05 when GD is compared with HC.

### 3.2 Comparison of levels of 6 serum cytokines between disease and HC groups

**Figure 1:**
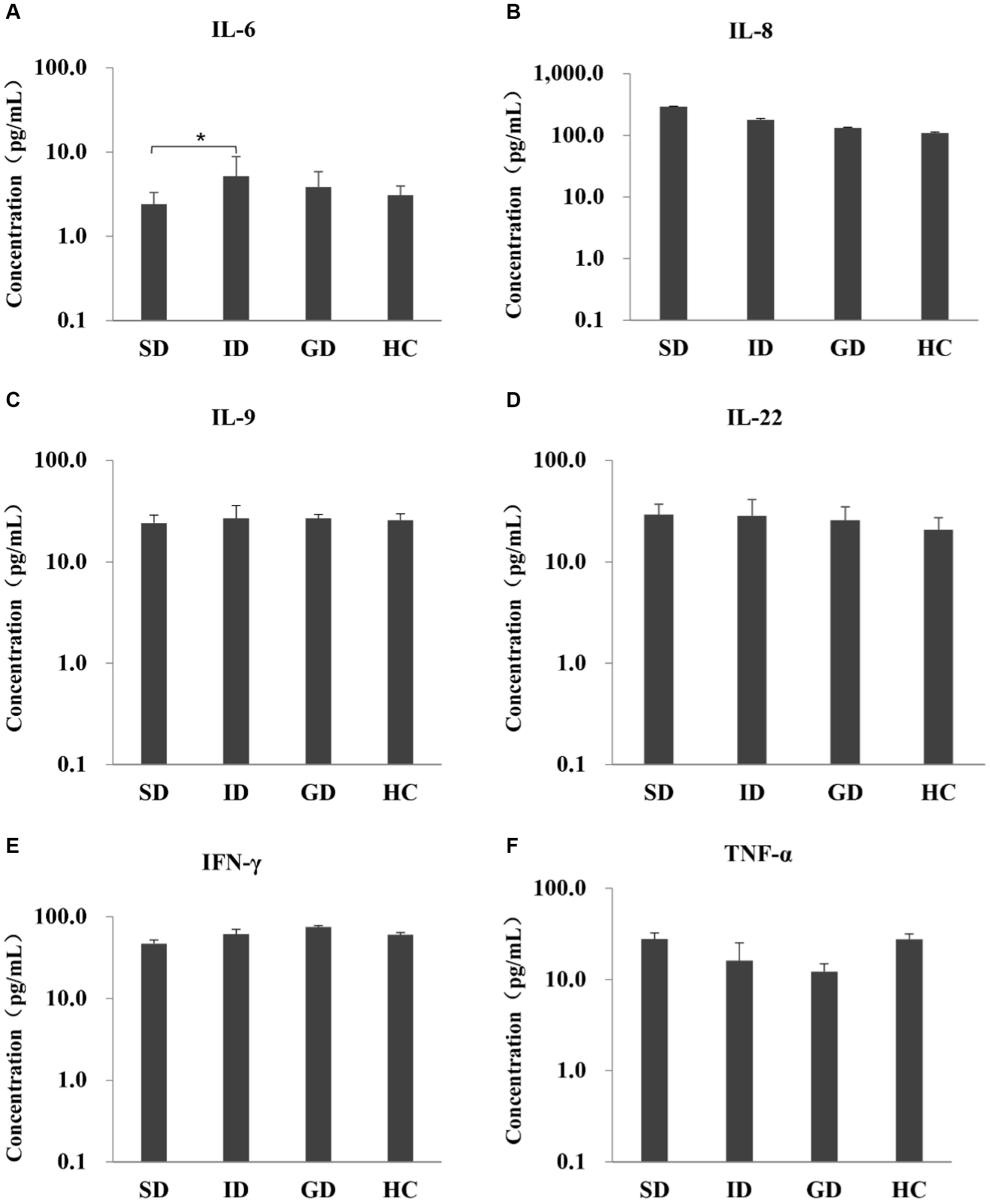
Evaluate the immune function of the disease and the HC groups by measuring the levels of six serum cytokines.

Among the 6 cytokines examined in this study, the serum level of IL-6 was highest in the ID group, with an average concentration of 5.20 pg./mL, which was significantly different from that in the SD group (*p* < 0.05), and there were no significant differences among other groups, as shown in Figure 1.

### 3.3 Analysis of community diversity among SD, ID and HC groups

**Figure 2:**
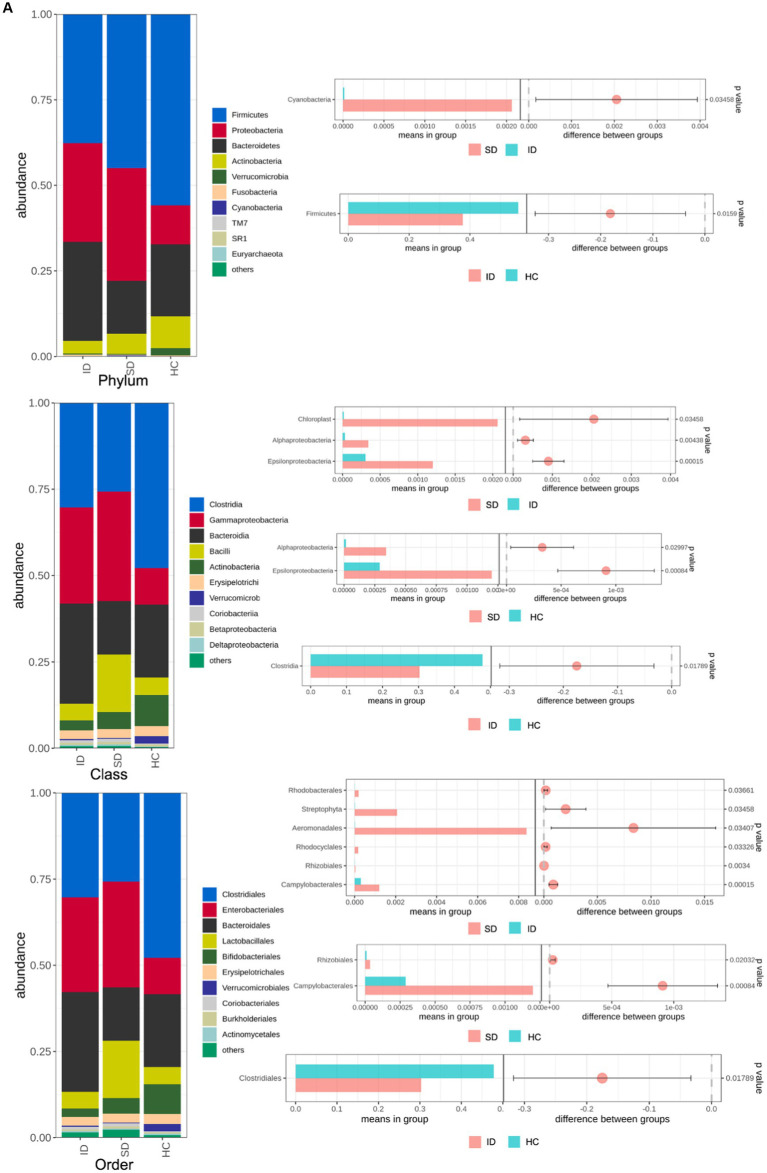

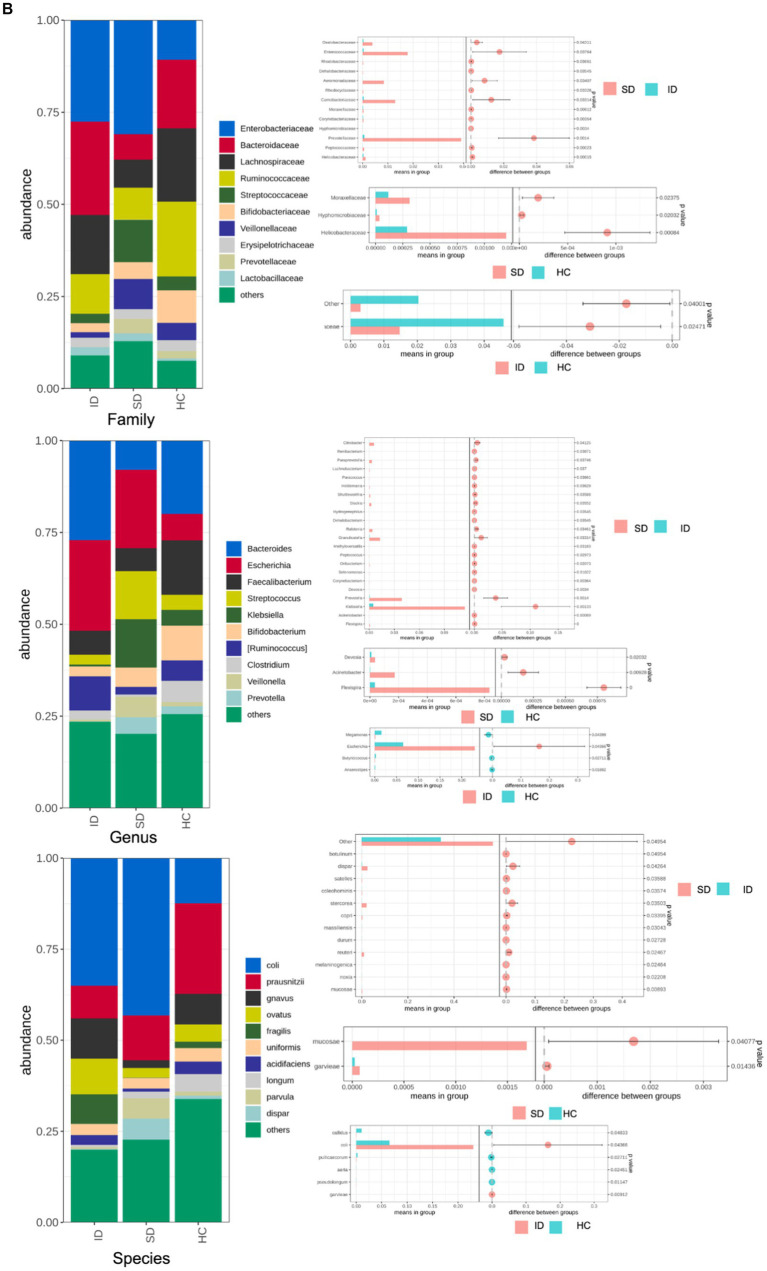

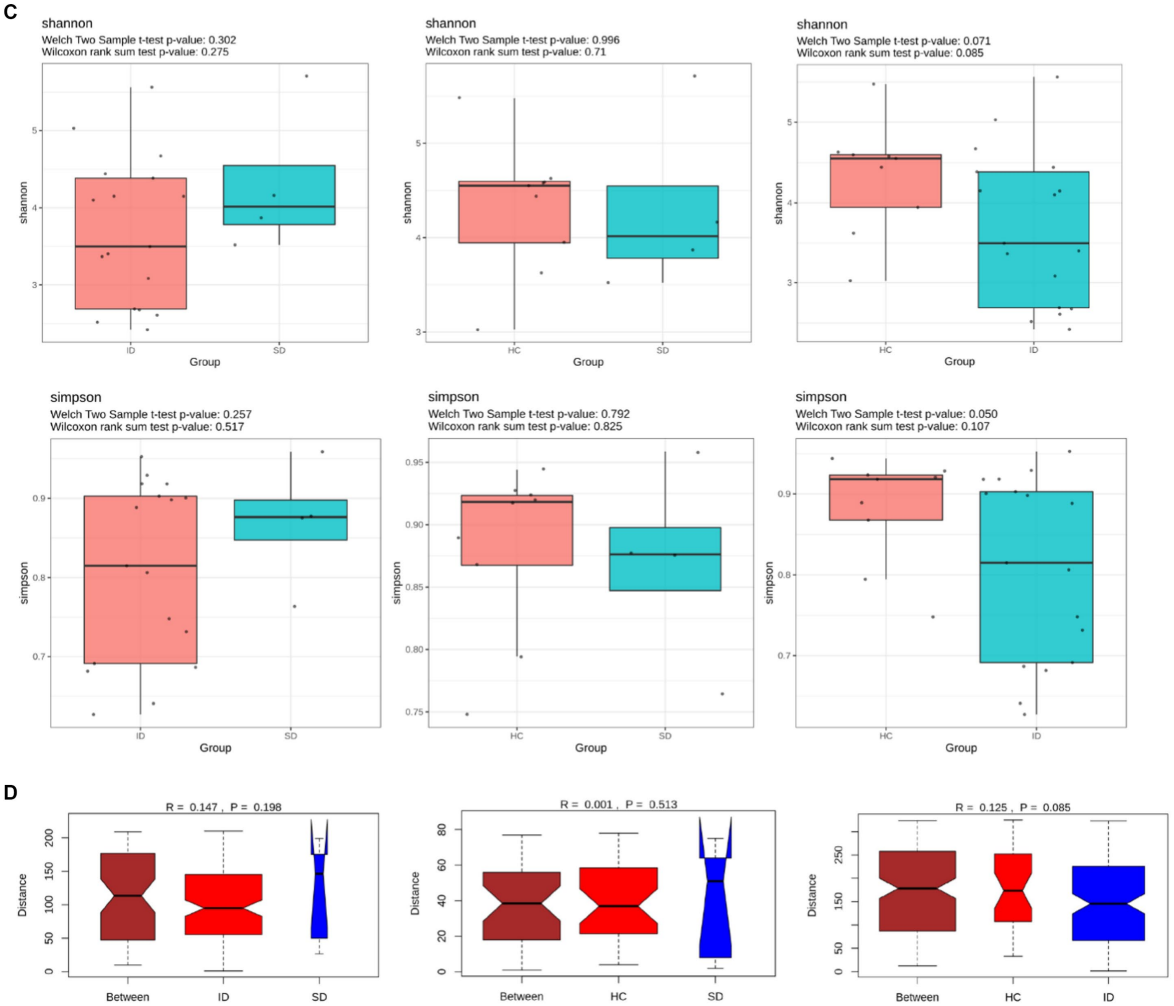


The species annotation results obtained from fecal samples of 4 patients in the SD group, 17 patients in the ID group, and 9 cases in the HC group revealed that the gut microbiota types in the SD and ID groups are more similar, with differences compared to the HC group. The gut microbiota in the SD, ID and HC groups primarily consisted of four predominant phyla: *Firmicutes, Bacteroidetes, Proteobacteria*, and *Actinobacteria*. The difference in *Firmicutes* between the ID and HC groups was significant. At the class level, *Clostridia, Gammaproteobacteria, Bacteroidia* and *Bacilli* were prevalent in both SD and ID groups, while *Clostridia, Gammaproteobacteria, Bacteroidia* and *Actinobacteria* were dominated in the HC group. Concerning the order level, *Clostridiales, Enterobacteriales, Bacteroidales* and *Lactobacillales* were dominant in both the SD and ID groups, whereas *Clostridiales, Enterobacteriales, Bacteroidales* and *Bifidobacteriales* were dominated in the HC group. The difference in *Clostridia* between the ID and HC groups was significant in class and order. On the family level, *Enterobacteriaceae, Bacteroidaceae, Lachnospiraceae* and *Ruminococcaceae* were more abundant in both ID and HC groups, while *Enterobacteriaceae* and *Streptococcaceae* were more abundant in the SD group. At the genus level, *Escherichia, Bacteroids* and *Ruminococcus* were more prevalent in the ID group, whereas *Escherichia, Streptococcus* and *Klebsiella* were more abundant in the SD group, and *Bacteroids, Faecalibacterium* and *Bifidobacteria* were more prominent in the HC group (Figures 2A,B). Furthermore, we conducted through α diversity analysis (Figure 2C) on the community diversity within the sample, using Shannon and Simpson indices to characterize the richness and evenness of microbial communities, indicated no significant differences between any two groups among the SD, ID and HC groups (*t*-test and nonparametric rank-sum test, *U* test, *p* > 0.05 for all; Figure 2C). Simultaneously through β diversity (Figure 2D) analyzed the differences in community structure between groups, and ANOSIM analysis was used to test whether the differences between groups were significantly greater than those within groups. We conducted a significance test for the differences between groups based on the rank of Bray–Curtis distance values. As determined by Bray–Curtis distance values (ANOSIM analysis, *p* > 0.05 for all), there were no significant distinctions among microbial communities between any two groups among the SD, ID and HC groups (Figure 2D).

Comparison of microbial communities. **(A,B)** The gut microbiota in SD, ID and HC groups are abundant at the levels of phylum, class, order, family, genus and species. And *t*-tests were conducted on different classification levels to obtain *p*-value, which was corrected using the FDR method. We selected only microbial communities with a *p* < 0.05 for each classification level to draw a forest map. **(C)** Comparison of α-diversity (Shannon and Simpson indices) between any two groups among SD, ID and HC groups. **(D)** Comparison of β-diversity (determined by Bray–Curtis distance values) between any two groups among SD, ID and HC groups.

### 3.4 Analysis of microbial composition in SD, ID and HC groups and its correlation with clinical characteristics

#### 3.4.1 Analysis of microbial composition

**Figure 3:**
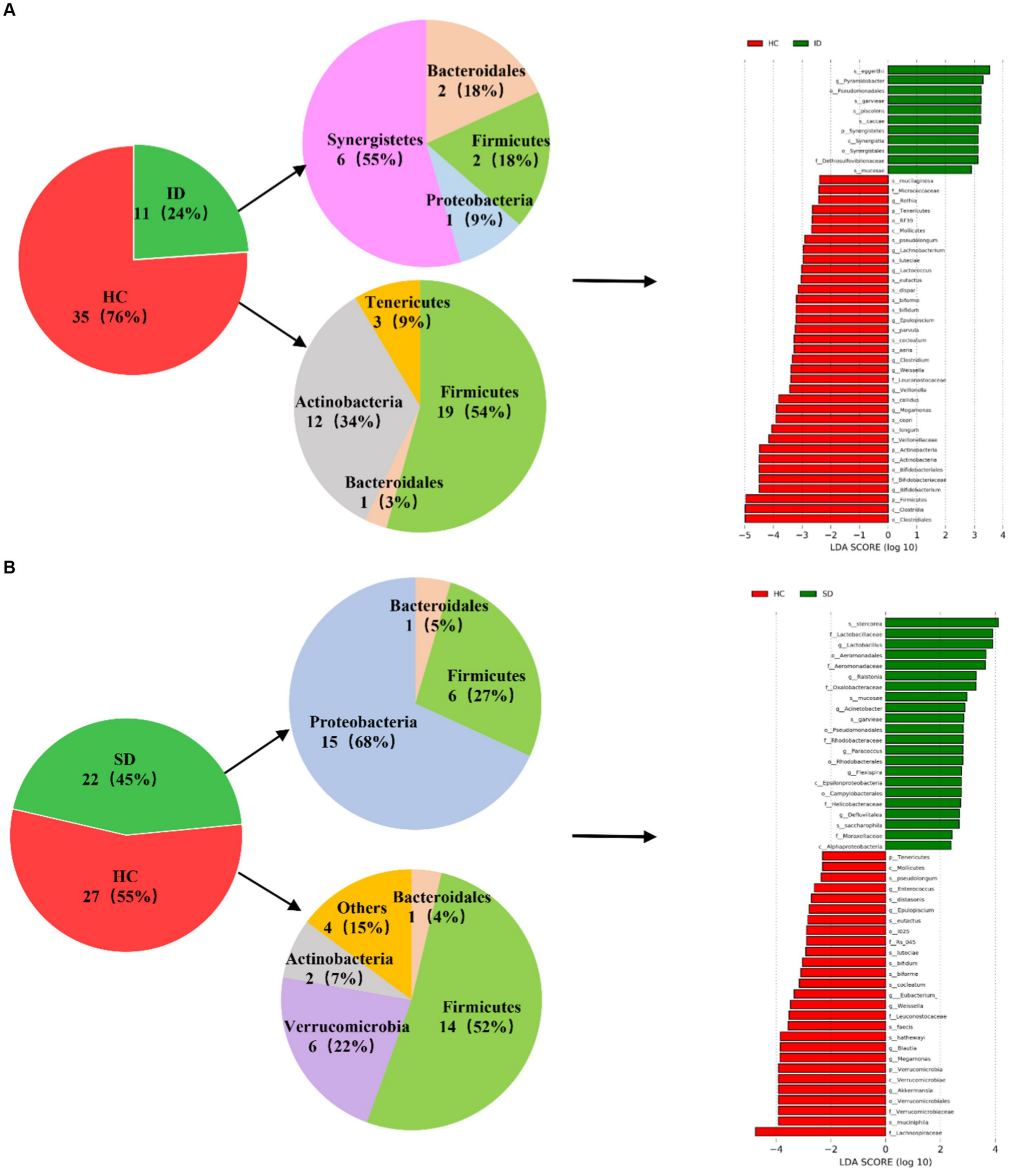
Analysis of microbial communities contributing significantly to intergroup differences. **(A)** Differences in species abundance between ID and HC groups. **(B)** Differences in species abundance between SD and HC groups.

As depicted in Figure 3, a comparative analysis between the HC group and both the ID and SD groups, respectively, involved the identification of species significantly contributing to intergroup distinctions and species exhibiting notable differences in abundance across different groups (as indicated by the LDA score), according to the results of LEfSe analysis. The results illustrated in Figure 3A revealed an enrichment of 6 species classified under the *phylum Synergistetes*, including *Pyramidobacter, Piscolens, Synergistetes, Synergistia, Synergistales* and *Dethiosulfovibrionaceae*, within the ID group. Conversely, 19 species from *Firmicutes* and 12 species from *Actinobacteria* exhibited enrichment in the HC group. It was indicated that the SD group was accompanied by an enrichment of 15 species associated with *Proteobacteria* and 6 species from *Firmicutes*, while the HC group demonstrated enrichment in 14 species from *Firmicutes* and 6 species from *Verrucomicrobia* (Figure 3B). It can be seen that the microbial community of the HC group exhibits higher taxonomic diversity.

#### 3.4.2 Correlation of microbial communities and clinical characteristics in SD, ID and HC groups

**Figure 4:**
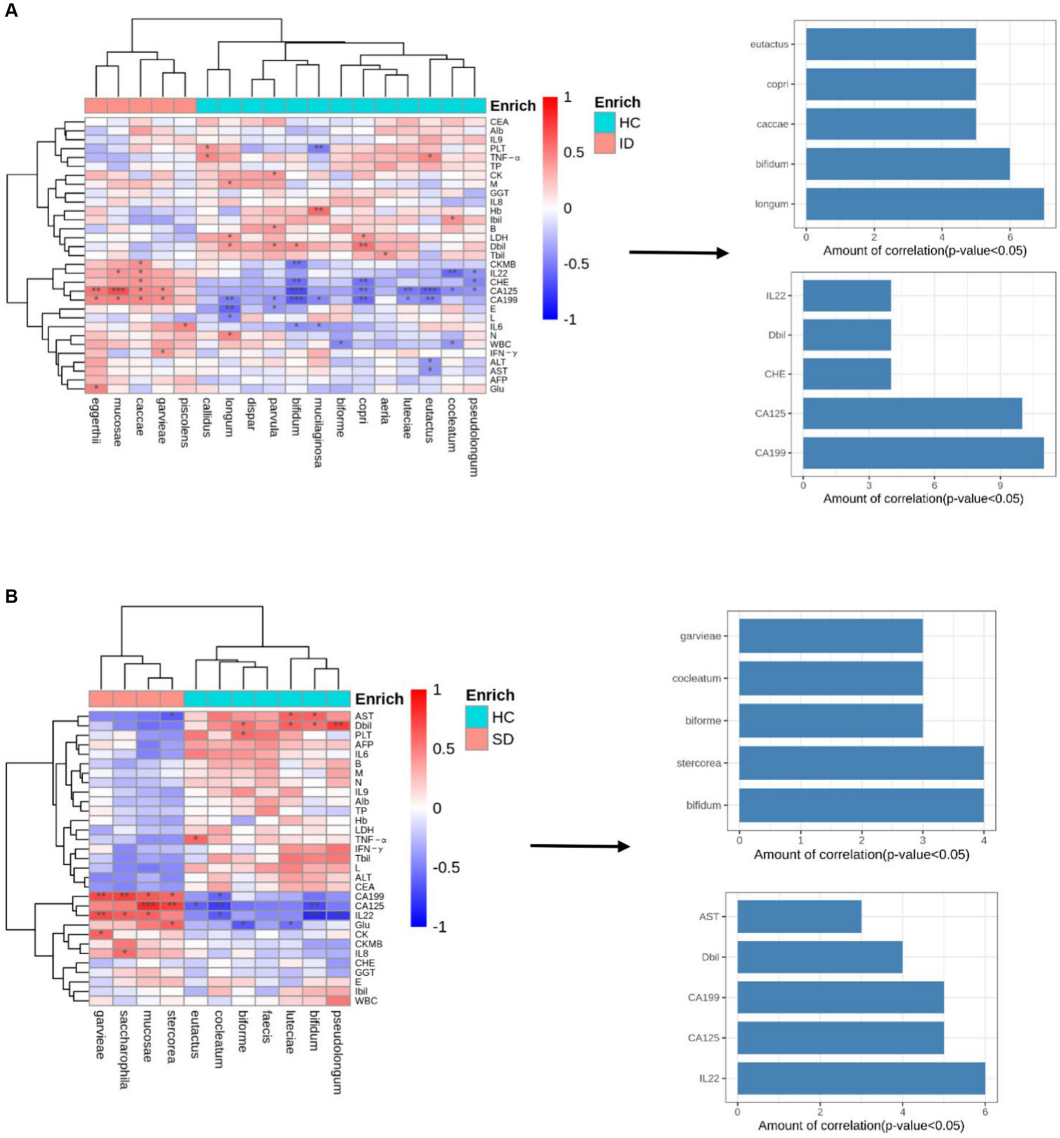
Association of microbial communities with clinical characteristics. **(A)** Correlation matrix of species and clinical parameters in the ID group vs. HC group. Red indicates a positive correlation, while blue indicates a negative correlation. *, ** and *** represent *p* < 0.05, *p* < 0.01, and *p* < 0.001, respectively; the number of significant correlations for the 5 species with the most frequent correlations and 5 clinical characteristics with the most frequent correlations (only the top five are listed). **(B)** Correlation matrix of species and clinical parameters in the SD group vs. HC group. Red indicates a positive correlation, while blue indicates a negative correlation. *, ** and *** represent *p* < 0.05, *p* < 0.01, and *p* < 0.001, respectively; the number of significant correlations for the 5 species with the most frequent correlations and 5 clinical characteristics with the most frequent correlations (only the top five are listed).

In the present study, Spearman correlation analysis was employed to examine the relationship between clinical attributes and species abundance in the ID group versus the HC group, as well as the SD group versus the HC group. The analysis comparing the ID group with the HC group (illustrated in Figure 4A) revealed that most of the gut microbes enriched in the ID group significantly exhibited positive correlations with 15 clinical characteristics, including creatine kinase-MB (CKMB), interleukin-22 (IL-22), CHE, CA125, CA199, E, L, IL-6, N, white blood cells (WBC), IFN-γ, alanine aminotransferase (ALT), aspartate aminotransferase (AST), AFP and glucose (Glu), among which CA125 and CA199 show a strong positive correlation. Conversely, they displayed negative correlations with 16 other clinical characteristics. In contrast, the HC group displayed the opposite pattern, in which the enriched microbes were significantly negatively correlated with the aforementioned 15 clinical characteristics and positively correlated with the other 16 clinical characteristics. Among them, the three indicators CHE, CA125 and CA199 showed a strong negative correlation, while DBIL showed a strong positive correlation. Similarly, in the SD and HC groups (Figure 4B), most gut microbes enriched in the SD group exhibited significantly positive correlations with 12 clinical characteristics, with CA125, CA199 and IL-22 showing a strong positive correlation, while demonstrating negative correlations with 19 additional clinical characteristics. Conversely, in the HC group, the microbes that were enriched displayed a contrasting pattern, in which they were significantly negatively correlated with the aforementioned 12 clinical characteristics, with CA125 and IL-22 showing a strong negative correlation, and positively correlated with other 19 clinical characteristics, with DBIL showing a strong positive correlation.

Among 46 species analyzed in the ID and HC groups, the species “*longum*” exhibited the strongest correlation with clinical characteristics (n = 7, positive correlation: M, LDH, DBIL and N; negative correlation: CA199, E and L, *p* < 0.05), followed by “*bifidum*” (*n* = 6, positive correlation: DBIL; negative correlation: CKMB, CHE, CA125, CA199 and IL-6, *p* < 0.05), “*copri,*” “*caccae*,” and “*eutactus*” (*n* = 5, *p* < 0.05, Figure 4A). Among them, LDH and DBIL are positively correlated with *copri*, while CHE, CA125 and CA199 are negatively correlated; CKMB, IL-22, CHE, CA125 and CA199 are positively correlated with *caccae*; TNF-α is positively correlated with *eutactus*, while CA125, CA199, ALT and AST are negatively correlated; Notably, only “*caccae*” was enriched in the ID group. Among 49 species analyzed in the SD and HC groups, “*bifidum*” and “*stercorea*” displayed the strongest correlation with clinical characteristics (*n* = 4, *p* < 0.05). Among them, AST and DBIL are positively correlated with *bifidum*, while CA125 and IL-22 are negatively correlated; Glu, CA125 and CA199 are positively correlated with *stercorea*, while AST is negatively correlated, followed by “*biforme*,” “*cocleatum*” and “*garvieae*” (*n* = 3, *p* < 0.05, Figure 4B); “*stercorea*” and “*garvieae*” were enriched in the SD group. Importantly, in both the ID and SD groups, the most pronounced correlation was identified between CA125/CA199 and bacterial species (ID group: *n* = 10/11, *p* < 0.05; SD group: *n* = 5/5, *p* < 0.05), followed by IL-22 in the SD group (*n* = 6, *p* < 0.05).

## 4 Discussion

The human microbiota and its host maintain a sophisticated symbiotic relationship, exerting significant impacts on host health. The balance and stability of the gut microbiota play a pivotal role in preserving human health. Dietary and healthy lifestyle habits are considered principal regulators of the gut microbiome, both in the short-term and long-term. Common clinical interventions to regulate the microbiome include microbiome modulation, fecal microbiota transplantation (FMT) and bacterial engineering (Lu et al., 2022). Furthermore, in addition to a well-balanced diet, supplementation with plant extracts and increased physical activity can also effectively regulate the gut microbiota (Zhou et al., 2023). Gastrointestinal diseases can be triggered by diverse factors, mainly manifesting with multiple symptoms, such as nausea, vomiting and diarrhea. Severe symptoms, including fever and dehydration, can also develop, potentially leading to carcinogenic changes. Thus, early prevention and treatment are imperative. However, conventional diagnostic methods as gastroscopy or tissue biopsy are invasive, expensive, painful and risky. These factors often hinder patients from undergoing these procedures, potentially delaying diagnosis and treatment (Dai et al., 2019; Wong and Yu, 2023).

The human gastrointestinal tract, in contrast to other body parts, hosts a substantial microbial community. Gut microbiota composition may vary with factors, such as age, environmental influences (e.g., medication use) and anatomical locations. These microbial communities play a vital role in human nutrient processing, metabolism and immunity (Bouskra et al., 2008). Notably, the overall diversity of human gut microbiota is constantly changing throughout life, as individuals age, the diversity of intestinal microbes decreases (Lynch and Pedersen, 2016), accompanying by a reduction in proportions of *Firmicutes* and *Actinobacteria* and an increase in the abundance of *Proteobacteria*. The diversity of gut microbiota in the elderly can significantly vary due to individual differences (Maynard and Weinkove, 2018; Adak and Khan, 2019; Shi et al., 2022). In the present study, significant differences were found in clinical characteristics among the disease groups versus the HC group. These differences pertained to age, CA125, CA199, AFP, TBIL and IBIL. We detected six cytokines closely related to the gut microbiota or its metabolites, hoping to further explore the serum immune function of patients with gastrointestinal diseases. The expression of inflammatory cytokines IL-6, IL-8 and TNF-α is related to the metabolism of dietary nutrients involved in intestinal microbiota. The metabolic end products trimethylamine N-oxide (TMAO) and short-chain fatty acids affect inflammation and immune response by affecting the expression of various receptors, signaling and apoptosis genes, and lead to the expression of these three inflammatory cytokines. IL-22 is also affected by short-chain fatty acids (Liu et al., 2017; Li et al., 2022). Similarly, the upregulation of IFN-γ is influenced by inosine, a purine metabolite of *Akkermansiamucophila* and *Bifidobacterium pseudolongum*, which activates immune cells through the inosine-A_2A_R-cAMP-PKA signaling pathway and stimulates phosphorylation of cAMP response element binding protein (pCREB). Thereby upregulating IFN-γ transcription (Mager et al., 2020). Interestingly, valeric acid and butyric acid also promote the expression of effector molecules such as IFN-γ and TNF-α (Luu et al., 2021). Then, IL-9 is a pleiotropic cytokine that affects a variety of cells, and IL-9 and its receptors contribute to the pathogenesis of ulcerative colitis, which can further develop into cancer if left untreated (Matusiewicz et al., 2017). Among the six cytokines examined, IL-6 exhibited the highest serum level in the ID group and demonstrated a significant difference compared to the SD group (*p* < 0.05). Research has indicated that IL-6 level significantly increased in patients with CRC and IBD. However, treatment with *Bifidobacterium* and mixed *lactic acid bacteria* reduced IL-6 level (Li et al., 2022; Zhong et al., 2022). Imbalance of gut microbiota can lead to activation of tumor associated macrophages (TAMs), thereby promoting the secretion of IL-6. IL-6 accelerates the development of CRC by promoting epithelial mesenchymal transition (EMT) formation (Wan et al., 2018). Notably, the release of IL-6 is also reported to be associated with the presence of diacyl phosphatidylethanolamine on the cell membrane of *Akkermansiamuciniphila (Akk bacterium)* (Bae et al., 2022). A growing body of evidence suggested that an imbalance in the human gut microbiota is closely correlated with the development and progression of gastrointestinal diseases. Consequently, it is necessary to conduct cross-sectional cohort studies on the relationship between gut microbiota, serum immunology and clinical characteristics to analyze the differences in gut microbiota in patients with gastrointestinal diseases, and explore the correlation between gut microbiota and clinical characteristics. The in-depth analysis of this study will provide insights into the pathogenesis of gastrointestinal disease patients.

The intestinal flora typically includes six phyla: *Firmicutes, Bacteroidetes, Actinobacteria, Proteobacteria, Fusobacteria* and *Verrucomicrobia*, with *Firmicutes* and *Bacteroidetes* as the predominant species (Manichanh et al., 2012). Consistent with the findings of this study, the gut microbiota of the SD, ID and HC groups were mainly composed of *Firmicutes*, *Proteobacteria*, *Bacteroidetes* and *Actinobacteria*, but gradually showed differences at the levels of class, order, family, genus and species (Figures 2A,B). As in adults, 60–70% of the gut microbiome is stable, but the degree of stability varies among different phyla (Faith et al., 2013). When the gut microbiota becomes imbalanced, its characteristics are changes in composition or abnormal fluctuations in quantity (He et al., 2023). This study was conducted through α diversity analysis (Figure 2C) and β diversity analysis (Figure 2D), we found that there were no significant differences in the diversity of α and β between the three groups, but there were significant differences in the types and quantities of microorganisms at different taxonomic levels (Figures 2A,B). For example, there are significant differences in *Firmicutes* and *Clostridia* between the ID group and the HC group. The reason is that the substances metabolized at different anatomical sites are different, resulting in different types of gut microbiota in different parts of the gastrointestinal tract (Flint et al., 2012; de Vos et al., 2022). And in various diseases, including irritable bowel syndrome (IBS), abnormalities in the gut microbiota can also lead to dysbiosis of the gut microbiota (Konig and Brummer, 2014). Compared with the HC group, the mucosal and fecal microbial diversity of IBS patients decreased after infection (Sundin et al., 2015). Existing research has consistently shown differences in gut microflora composition between IBD patients and HCs. Specifically, IBD patients tend to exhibit a decrease in the proportion of *Bacteroidetes* and *Firmicutes*, accompanied by an increase in *γ-proteobacteria* (Morgan et al., 2012). Compared with HC, the microbial community of CRC patients is mainly composed of pathogenic bacteria related to metabolic disorders, while the abundance of probiotics (such as butyrate producing bacteria) is reduced, such as *Rothia*, *Clostridium*, *Fecal* and *Bifidobacterium*, which are usually lower in abundance (Yu et al., 2017; Thomas et al., 2019). Main bacteria present in CRC patients include *E. faecalis, E. coli, B. fragilis, S. bovis, F. nucleatum* and *H. pylori* (Dai et al., 2019). Among them, *F. nucleatum*, which is enriched in CRC, exhibited a close relationship with CRC-associated *Aspergillus rambellii*. This co-enrichment suggests that combined biomarkers involving both fungi and bacteria have more potential as diagnostic markers for CRC than pure bacterial groups (Lin et al., 2022). In addition, the gut microbiota can interact with CRC cancer cells, affecting the tumor microenvironment, thereby enhancing tumor invasiveness (Chen et al., 2022). Through synergistic inflammatory tumor promotion mechanisms, it promotes invasive mesenchymal transition and facilitates distant metastasis of invasive CRC in susceptible tissue environments (Slowicka et al., 2020), such as colorectal cancer liver metastasis (CRLM) (Wei et al., 2024). In the present study, when comparing the HC group with the ID group, significant differences were found in 46 species, with 35 belonging to the HC group and 11 to the ID group. The primary contributors to the HC group were *Firmicutes* and *Actinobacteria*, while *Synergistetes* played a predominant role in the ID group. Among these 46 species, 13 exhibited higher abundances than the others, including *Clostridiales, Clostridia, Firmicutes, Bifidobacterium, Bifidobacteriaceae, Bifidobacterales, Actinobacteria, Actinomyces, Veillonellaceae, Longum, Copri, Megamonas* and *Callidus*. Notably, *Bifidobacterium* is known for its role in stimulating the immune system and is associated with worsening inflammatory status (Guigoz et al., 2008). Furthermore, the richness of intestinal flora in the HC group was higher versus that in the ID group. When comparing the HC and SD groups, significant differences were identified in 49 species, with 27 belonging to the HC and 22 to the SD groups. Among these 49 species, only *Lachnospiraceae* in the HC group displayed a higher abundance than others. Therefore, from the perspective of microbial quantity differences between the HC and SD groups, the variations were not substantial. However, after gastrectomy, gastric cancer (GC) patients experience a significant increase in specific aerobic bacteria (*Streptococcus I* and *Enterococcus*) and facultative anaerobic bacteria (*Escherichia coli*, *Enterobacterium* and *Streptococcus*) due to increased intestinal oxygen content and translocation of oral microbiota (Palleja et al., 2016; Ilhan et al., 2017; He et al., 2023). So, it is essential to consider the distribution of species richness within the gut microflora, where the HC group maintained a higher diversity compared with the SD group. By comparing the species abundance of these three groups, we found that the microbial community of the HC group exhibited high taxonomic diversity, high microbial gene richness, and stable core microbial community, which is consistent with previous research (Fan and Pedersen, 2021). The significance of exploring the differences in the composition of gut microbiota between patients with gastrointestinal diseases and healthy individuals is that these results may contribute to the development of strategies for restoring microbial profiling in patients with gastrointestinal diseases. Microbial communities are powerful targets for treating gastrointestinal diseases. A detailed analysis of the diversity and richness of gut microbiota in patients with gastrointestinal diseases will provide new strategies for future prevention of gastrointestinal diseases and potential clinical applications of gut microbiota in treatment, including as anti-tumor drugs and affecting the efficacy of immunotherapy.

Moreover, it was found that some clinical characteristics (e.g., CA125, CA199, Glu, CKMB, IL-22, etc.) in the ID and SD groups were significantly positively correlated with enriched gut species and negatively correlated with the HC group. CA125 and CA199 are major tumor markers related to gastrointestinal cancer. Gastrointestinal cancer cells produce CA199 and CA125 proteins, which enter the blood stream and can be identified through marker tests. The positivity rates of CA125 and CA199 are relatively low in early-stage gastric cancer (Feng et al., 2017), the positivity rates of CA199 and CA125 in stages III/IV gastric cancer are higher than those in stages I/II, and preoperative serum positivity for both is associated with poor prognosis; they can be utilized to predict recurrence or metastasis of gastric cancer (Zhou et al., 2018). In the present study, there were significant differences in CA125 and CA199 levels when comparing the ID group and SD group with the HC group, and they were significantly positively correlated with *Firmicutes’ Garvieae, Mucosae*, and *Saccharophila*, in which *Bacteroidetes’ Eggerthii, Caccae* and *Stercorea* were among the enriched gut species. This indicates that combined detection of clinical characteristics, such as CA125 and CA199 along with microbial analysis can improve diagnostic efficiency for gastrointestinal diseases. Similarly, Glu and CKMB are frequently utilized clinical indicators. It was revealed that Glu and CKMB in the ID and SD groups showed no significant difference compared with HC group, while they were significantly positively correlated with *Bacteroidetes’ Eggerthii, Caccae, Stercorea* and *Firmicutes’ Saccharophila*. The gastrointestinal tract plays a crucial role in digesting sugars from food. For instance, *Bacteroides* species located within the colon are responsible for sugar uptake (Martens et al., 2008), while pathogenic *Enterobacteriaceae* can also utilize sugars and amino acids present within the intestine (Ducarmon et al., 2019). The cytokine IL-22 is of paramount importance in the intestinal defense mechanisms against microbiota. In the present study, the results showed that IL-22 level within the ID and SD groups did not significantly differ from that in the HC group. However, IL22 exhibited a significantly positive correlation with specific species within the *Firmicutes phylum*, namely *Garvieae, Mucosae* and *Saccharophila*, as well as with *Bacteroidetes’ Caccae* among the enriched species. Consistent with this study, *Lactobacillus plantarum* belonging to the *Firmicutes* can induce NK cells to secrete IL-22 (Suzuki, 2013). Existing research suggests that certain bacterial products, such as LPS, Pam2 and Pam3, can stimulate the production of IL-22 within T cells, and this response is further enhanced by macrophage-derived TNFα-induced expression (Klooster et al., 2021). Furthermore, short chain fatty acids (SCFAs) can promote IL-22 production in CD4 T cells and ILCs through GPR41 and inhibition of histone deacetylase (Yang et al., 2020). It is noteworthy that the present study revealed correlations between gut microbiota and specific clinical characteristics among patients suffering from gastrointestinal diseases. However, it is crucial to notice that there is currently limited research in this area. As a result, this research findings will carry valuable clinical reference significance and provide additional opportunities and possibilities for the diagnosis and treatment of patients with gastrointestinal diseases.

Therefore, comprehending the association and underlying mechanisms connecting clinical characteristics, serum immunology, and gut microbiota in individuals afflicted by gastrointestinal conditions can significantly enhance diagnostic and therapeutic approaches. This insight can be pivotal in exploring the potential role of microbial communities within the stomach and intestines. Notably, gastric and intestinal diseases, while closely intertwined, exhibit distinctive characteristics. Tailoring interventions for regulating gut microbiota to influence disease progression holds promise in averting and mitigating the incidence of gastrointestinal disorders, leading to develop personalized multimodal therapeutic strategies. Given the limited sample size in this study, future investigations should encompass larger cohorts to more deeply assess into the intricate interplay among clinical attributes, serum immunology, and gut microbiota in patients with gastrointestinal diseases, but the results of the present work may constitute a good starting point for this purpose. And we should focus on exploring new ideas for prevention, early intervention and treatment of gastrointestinal diseases by intervening in the microbial community of antibiotics or blocking the production of bacterial components. The future research will shed light on the underlying mechanisms driving these conditions.

## 5 Conclusion

In conclusion, this study, employing different analyses of clinical characteristics, serum immunology and gut microbiota in patients with gastrointestinal diseases, revealed a close association between imbalances in human gut microbiota and the onset and advancement of gastrointestinal diseases. Notably, the gut microbiota in the HC group exhibited a greater abundance versus the ID and SD groups, and there were significant differences in microbial species among the three groups at different classification levels. Additionally, a discernible correlation was established between certain clinical characteristics, including CA125, CA199, IL-22, among others and gut microbiota among patients with gastrointestinal diseases. Therefore, in the diagnosis and management of gastrointestinal diseases, combining clinical features with serum immunology and gut microbiota will greatly promote the diagnosis and treatment of gastrointestinal disease patients by clinical doctors, especially in terms of personalized multi-mode precise treatment strategies. In addition, the results of this study will provide new insights into the pathogenesis of gastrointestinal diseases in patients, and provide new ideas for the prevention and early intervention of gastrointestinal diseases.

## Data availability statement

The data presented in the study are deposited in the NCBI SRA repository, accession number PRJNA1073875.

## Ethics statement

The studies involving humans were approved by Medical Research Ethics Committee of Jiaying University. The studies were conducted in accordance with the local legislation and institutional requirements. The participants provided their written informed consent to participate in this study.

## Author contributions

HC: Conceptualization, Methodology, Writing – original draft, Writing – review & editing. YZ: Investigation, Methodology, Writing – review & editing. GC: Data curation, Formal analysis, Writing – review & editing. WG: Resources, Writing – review & editing. YY: Funding acquisition, Project administration, Writing – review & editing.
